# Tumor burden monitoring using cell-free tumor DNA could be limited by tumor heterogeneity in advanced breast cancer and should be evaluated together with radiographic imaging

**DOI:** 10.1186/s12885-017-3185-9

**Published:** 2017-03-22

**Authors:** José Angel García-Saenz, Patricia Ayllón, Marion Laig, Daniel Acosta-Eyzaguirre, Marta García-Esquinas, Myriam Montes, Julián Sanz, Miguel Barquín, Fernando Moreno, Vanesa Garcia-Barberan, Eduardo Díaz-Rubio, Trinidad Caldes, Atocha Romero

**Affiliations:** 1 Medical Oncology Department, Hospital Clínico San Carlos, Madrid, Spain; 2 Thermo Fisher Scientific, Waltham, Massachusetts USA; 3 Medical Oncology Department, Centro Integral Oncológico Clara Campal HM hospitals, Madrid, Spain; 4 Radiology Department, Hospital Clínico San Carlos, Madrid, Spain; 5 Nuclear Medicine Department, Hospital Clínico San Carlos, Madrid, Spain; 6 Pathology Department, Hospital Clínico San Carlos, Madrid, Spain; 7 Medical Oncology Department, Hospital Universitario Puerta de Hierro Madrid, 28222 Majadahonda, Madrid Spain

**Keywords:** Breast cancer, dPCR, PIK3CA, cfDNA

## Abstract

**Background:**

Accurate measurement of tumor burden in breast cancer disease is essential to improve the clinical management of patients. In this study, we evaluate whether the fluctuations in the fraction of PIK3CA mutant allele correlates with tumor response according to RECIST criteria and tumor markers quantification.

**Methods:**

Eighty six plasma samples were analyzed by digital PCR using Rare Mutation Assays for E542K, E545K and H1047R. Mutant cfDNA and tumor markers CA15-3 and CEA were compared with radiographic imaging.

**Results:**

The agreement between PIK3CA mutation status in FFPE samples and circulating tumor DNA (ctDNA) was moderate (K = 0.591; 95% IC = 0.371–0.811). Restricting the analysis to the metastatic patients, we found a good agreement between PIK3CA mutation status assessed in liquid and solid biopsy (K = 0.798 95%; IC = 0.586–1). ctDNA showed serial changes with fluctuations correlating with tumor markers 15.3 and CEA in 7 out of 8 cases with Pearson correlation coefficients ranging from 0.99 to 0.46 and from 0.99 to 0.38 respectively. Similarly, fluctuations in the fraction of PIK3CA mutant allele always correlated with changes in lesion size seen on images, although in two cases it did not correlate with treatment responses as defined by RECIST criteria.

**Conclusion:**

oncogenic mutation quantification in plasma samples can be useful to monitor treatment outcome. However, it might be limited by tumor heterogeneity in advanced disease and it should be evaluated together with radiographic imaging.

**Electronic supplementary material:**

The online version of this article (doi:10.1186/s12885-017-3185-9) contains supplementary material, which is available to authorized users.

## Background

Tumor biopsies have been and currently are the keystone for biomarker testing [1]. However, some limitations of tumor biopsies are still significant, such as difficulties in obtaining tissue samples, the invasiveness of the process or tumor heterogeneity, which might compromise the efficacy of targeted-therapies, based on a single biopsy [2, 3].

Tissue cells release circulating free DNA (cfDNA) into the bloodstream. Specifically, tumor-derived DNA (ctDNA) can be detected in plasma samples by identifying specific genetic alterations that tumors harbor, using highly sensitive technologies, such as digital PCR (dPCR), with the advantage that the aforementioned limitations can be overcome. In addition, quantification of tumor-specific mutations in liquid biopsies has been shown to correlate with tumor burden and there is growing evidence suggesting that ctDNA monitoring can predict treatment outcome [4–9].

At present, tumor response to treatment is commonly evaluated according to RECIST criteria. However, several studies have pointed out the limitations that radiology assessment may have when assessing tumor response, especially in estrogen receptor (ER) positive breast cancer patients [10–13].

The PIK3CA gene encodes the p110α catalytic subunit of PI3K protein, and is mutated in over one-third of breast cancer cases [14]. Specifically, 40% of hormone dependent cancers harbor activating mutations in PIK3CA [14–17], making this gene an attractive candidate for tumor monitoring by tumor-specific mutation quantification in plasma samples. Mutations occur predominantly in the helical domain (generally E542K and E545K) and the kinase domain (generally H1047R) and account for 70% of all PIK3CA mutations in breast tumors [14–17]. Hotspot mutations have been shown to confer oncogenic features although the prognostic/predictive significance of PIK3CA mutations remains controversial.

In this study, we compare the clinical information provided by PIK3CA mutation quantification using array-based dPCR, in plasma samples from ER positive breast cancer patients, with tumor markers 15-3 and CEA and computed tomography (CT) scan assessments.

## Methods

### Study population

A total of 49 patients with estrogen receptor (ER) positive breast cancer were prospectively enrolled in the study after signing the appropriate informed consent. Patients agree to the publication of anonymized data. The study protocol was approved by the Hospital Clinico San Carlos Ethics Committee (internal code 13/383-E). Eligibility criteria comprised the following: women aged between 18 and 80 years with a pathologically confirmed diagnosis invasive breast carcinoma, clinical stage IIB-IIIB or IV and availability of the archived tissue for genotyping at the Hospital Clinico San Carlos Biobank. Blood samples were collected at the beginning of a treatment that the patient was going to initiate when included in the study. In 43 cases, the tumor specimen collected was the primary breast tumor; a biopsy of a FFPE metastatic breast cancer lesion was available in the remaining 6 patients. Information concerning demographics, clinicopathological features (stage, grade, estrogen and progesterone receptor status and HER2 status), courses of treatment(s), and vital status was obtained from the clinical and pathology reports (Additional file 1).

### Laboratory analysis

Eighty six blood samples were collected in BD Vacutainer® CPT™ Cell Preparation Tube with Sodium Heparin (containing a gel barrier). Hemolyzed samples were discarded for further analyses. Plasma was derived from blood samples by centrifugation within 3 hours after blood extraction. cfDNA was isolated from 3 ml of plasma using the QIAamp Circulating Nucleic Acid Kit (Qiagen, Valencia, CA), according to manufacturer’s instructions. cfDNA was eluted in 35 μL of the supplied elution buffer. The eluate was reloaded in the column and eluted by maximum speed centrifugation in order to increase the amount of cfDNA. Samples were then analyzed by digital PCR, using Rare Mutation Assays^1^ for E542K (AHD2BSD), E545K (AHABHHX) and H1047R (AHPAVCD) on QuantStudio® 3D Digital PCR System (Applied Biosystems, South San Francisco, CA). For the dPCR reaction, 8 ul of cfDNA was mixed with 0.5 ul of the aforementioned 40X TaqMan assays and 10 ul of 2x QuantStudio 3D Master Mix, in a 20ul reaction volume. Subsequently, 15 ul were loaded into QuantStudio 3D Digital PCR 20 K chips. The cycling conditions were as follows: initial denaturation at 96 °C for 10 min, followed by 40 cycles at 56 °C for 2 min, and 98 °C for 30s, a step of 72 °C for 10 min and finally samples were kept at 22 °C for at least 30 min. Chip fluorescence was read twice. Results were analyzed with QuantStudio® 3D Analysis Suite™ Cloud Software. The automatic call assignments for each data cluster where manually adjusted when needed. The analysis of dPCR data was performed blinded to the clinical and tumor information by two independent investigators (AR and ML). Double positive data points (green data points) where only considered when FAM data points where visualized. The result of the assay is reported as mutant allele frequency (MAF) which is defined as the ratio of mutant DNA molecules vs the sum of wild-type (wt) DNA molecules and mutant DNA molecules. Samples were considered as positive when MAF was greater than 0.15%. A wt control DNA was included in every run. All assays were validated using plasmid DNAs harboring the aforementioned the PIK3CA mutations (GeneArt, Thermo Fisher Scientific). Germline wt DNA from healthy donors was mixed with different mutant allele concentrations (i.e. 1%, 0.5%, 0.1%, 0.05%) in order to estimate the Limit of quantitation (LOQ) and limit of detection (LOD). LOD and LOQ were calculated based on the standard deviation of the response and the slope according to ICH Q2(R1) guideline (http://www.ich.org/products/guidelines/quality/article/quality-guidelines.html). The standard deviation of the response was calculated based on standard error of the y-intercept. Additionally, LOD and LOQ were estimated based on blank measurements. In this case, LOD was expressed as the average of MAF corresponding to the wt samples plus three standard deviation and LOQ was the average of MAF corresponding to the wt samples plus ten standard deviations. Two replicates were performed for all patients.

For the analyses of FFPE tumor and metastasis samples, a hemotoxylin and eosin-stained slide was reviewed by a pathologist to confirm the presence of tumor cells within the section. Overall, the percentage of tumor cells in the analyzed samples ranged from 80 to 90%. Subsequently, DNA was purified from four 5-mm-thick unstained FFPE sections according to the QIAamp DNA FFPE Tissue Kit (Qiagen, Valencia, CA) protocol.

The identification of PIK3CA mutations on FFPE samples was performed using COBAS® PIK3CA Mutation Test (Roche Molecular Systems, Branchburg, NJ). The test is intended to detect R88Q in exon 1, N345K in exon 4, C420R in exon 7, E542K, E545X (E545A, E545D,E545G, and E545K), Q546X (Q546E, Q546K, Q546L, and Q546R) in exon 9, and M1043I, H1047X (H1047L, H1047R, and H1047Y), and G1049R in exon 20 when the percent mutation is equal or greater than 5%. PIK3CA mutation status was further confirmed by the aforementioned TaqMan assays on the QuantStudio® 3D Digital PCR System. Conflicting samples were further amplified and sequenced. Primers and conditions used are available upon request. PCR products were then sequenced on the ABI 3130 genetic analyzer (Applied Biosystems, Foster City, CA), using the BigDye Terminator v1.1 Cycle Sequencing Reaction kit (Applied Biosystems, Foster City, CA) following the manufacturer’s protocol kit.

Serum samples were used to measure the levels of CA 15–3 antigen and CEA (Carcinoembryonic Antigen) using the high throughput immunoassay analyzer UniCelDxI 800 (Beckman Coulter), according to manufacturer’s instructions. In brief, the assay is a two-step sandwich immunoassay using direct, chemiluminescent technology.

### Response evaluation

Mammogram, ultrasound-based, magnetic resonance imaging (MRI), computed tomography (CT) and Positron emission tomography (PET)-CT tumor measurements were obtained, as clinically indicated. The clinical response was evaluated according to RECIST v1.1 criteria comparing radiological assessments. Complete response (CR) was defined as resolution of all palpable, visible or radiology abnormalities in the breast and regional lymph nodes. Partial response (PR) was defined as a decrease of ≥30% in the sum of the longest diameter in the breast and regional lymph nodes. Stable disease (SD) was assigned to patients who did not meet the criteria for CR, PR or PD. Clinical Progressive disease (PD) was defined as an increase of at least 20% in the sum of the longest diameter in the breast and regional lymph nodes or progression of other clinical manifestations of disease.

### Statistic analysis

Continuous variables are presented as means ± standard deviations (SD) and discrete variables as proportions. The weighted quadratic kappa coefficient values and the corresponding 95% confidence intervals (95% CI) were estimated in order to measure the strength of agreement between methodologies. The strength of agreement is judge to be minor when the К values are between 0.00 and 0.20; fair, 0.21 and 0.40; moderate, 0.41 and 0.60; good, 0.61 and 0.80 and nearly perfect, 0.81 and 1.00. Correlation between quantitative variables was evaluated using the Pearson correlation coefficient. The correlation among the quantitative variables was evaluated with simple linear regression analysis. *P* < 0.05 was considered to be statistical significance. The statistical analysis was performed using software R 3.0.1.

## Results

### Assay performance and agreement between methodologies

We report data from 49 patients with ER positive breast cancer undergoing therapy that were prospectively enrolled in the study. The pathological characteristics of study population are summarized in Table 1 and Additional file 1. The source of archival tissue sample was the primary tumor for 43 (87.8%) patients and biopsy of a distant metastasis for 6 (12.2%). DNA isolated from archival-tumor tissue samples was analyzed to identify PIK3CA somatic mutations. According to COBAS, among the analyzed tumors, 11 (22.4%) harbored the mutation H1047R, 5 (10.2%) harbored the E545K and 4 (8.1%) harbored the E542K. These are expected frequencies according to previously published data. Interestingly, we did not encounter any other less frequent PIK3CA mutation that could be detected by COBAS methodology. Subsequently, the three PIK3CA hot spot mutations were screened in FFPE derived samples using QuantStudio 3D system. The proportion of observed agreement between COBAS and QuantStudio3D system when assessing the PIK3CA mutation status in the archival tumor was 100% (K = 1) (Additional file 1).

**Table 1:** Clinicopathological features of the study population

*Clinicopathologic Characteristics*	
*Age at diagnosis (years)*	
*Median (range)*	62 (30–80)
*Histology (n (%))*	
*Ductal*	44 (90%)
*Lobular*	5 (10%)
*Histologycal grade (n (%))*	
*I and II*	39 (80%)
*III*	10 (20%)
*UICC stage (n (%))*	
*IIB*	6 (12%)
*IIIA + IIIB*	11 (22%)
*IV*	32 (65%)
*ER status (n (%))*	
*positive*	49 (100%)
*negative*	0 (0%)
*PR status(n (%))*	
*positive*	33 (67%)
*negative*	16 (33%)
*HER2 status (n(%))*	
*positive*	8 (16%)
*negative*	41 (84%)

*Abbreviations*: *ER* estrogen receptor, *PR* progesterone receptor, *HER2* erb-b2 receptor tyrosine kinase 2, *UICC* Union for International Cancer Control

Next, we evaluated the detection sensitivity of TaqMan assays. For sensitivity assays plasmid carrying E542K, E545K and H1047R mutations were mixed at different allele concentrations with wt DNA extracted from peripheral blood cells form healthy donors. Mutant allele frequencies correlated with the expected mutant allele frequencies in E542K assay, E545K assay and H1047R assay (Pearson’s correlation coefficient, 0.9985, 0.9966 and 0.9991 respectively). LOD for E542K, E545K and H1047R assays were 0.04, 0.05 and 0.03% respectively. LOQ for E542K, E545K and H1047R assays were 0.13, 0.15 and 0.08% respectively (Additional file 2). Additionally, wt DNA from healthy donors were used to evaluate the false positive signals. The mean of mutant allele fraction of wt DNA form healthy individuals was 0.01% for E542K assay, 0.00% for E545K assay and 0.01% for H1047R.

Finally, we evaluated the agreement between PIK3CA mutation status of FFPE samples and the matched plasma sample. The median time between blood collection at study entry and time that the tumor biopsy was obtained from the patient was 7 years (range = 0–15 years). Importantly, cfDNA from all plasma samples was successfully amplified. Representative plots for the three assays, performed on cfDNA, are displayed in Additional file 3. The ratio of mutant DNA molecules vs total DNA molecules ranged from 0.19 to 53.4% in the positive sample cases. The median of PIK3CA mutated copy number in positive samples was 1496 copies/ml (range: 63.3–92742).

Overall, we found a moderate agreement between PIK3CA mutation status in plasma and FFPE tumor samples (K = 0.591; 95% IC = 0.371–0.811). Specifically, we found that in 9 samples, the assay failed to detect on cfDNA the mutation identified in the tumor sample (data obtained from Additional file 1). However, we did not find any mutation in the plasma that was not present in the FFPE sample (sensitivity 55%, specificity 100%, positive predictive value 100%, and negative predictive value 76%). Restricting the analysis to the metastatic patients, we found a good agreement between PIK3CA mutation status assessed in liquid and solid biopsy (K = 0.798 95%; IC = 0.586-1) (data obtained Additional file 1). In this case, the cfDNA assay failed to detect the tumor mutation in 3 samples (sensitivity 77%, specificity 100%, positive predictive value 100%and negative predictive value 86%).

### Correlation with tumor markers

In 8 breast cancer patients with stage IV disease in which PIK3CA somatic mutation was identified in both FFPE and plasma samples, mutant cfDNA and tumor markers CA15-3 and CEA were quantified in a total of 27 serial plasma samples (Table 2). Circulating tumor DNA (ctDNA) was identified in 21 of the 27 plasma samples analyzed. Tumor markers CA15-3 and CEA levels were evaluated at all-time points in all 8 patients. Circulating tumor DNA showed serial changes with fluctuations correlating with tumor markers in 7 cases. In one case (from now on patient 3) changes in PIK3CA mutation quantification were in the opposite direction than tumor markers. In patients in which tumor markers CA15-3 and CEA and PIK3CA mutant DNA fluctuated in the same way, Pearson correlation coefficients ranged from 0.99 to 0.46 and from 0.99 to 0.38 respectively (mean 0.82 for CA 15–3 and 0.79 for CEA).

**Table 2:** ctDNA and tumor markers measurements in 8 breast cancer patients with stage IV disease

Patient	Tracked mutation	Sample	Target/Total	mutated copies/mL	CA 15.3 (U/ml)	CEA (ng/ml)	Figure
25	H1047R	March 07 2014	18.36%	54398	97	27	Additional file 4: Figure S1
		Oct 13 2014	0.11%	12.1	32.8	7.1	
		Nov 03 2014	0%	0	32.3	7.5	
44	H1047R	Aug 29 2014	1.78%	310.5	126	1.6	Fig. 1b
		Sep 17 2014	1.85%	975	997.2	5.2	
		Oct 23 2014	2.08%	1203.2	1219	11.6	
		Nov 19 2014	0.81%	102.9	487.3	4.8	
15	H1047R	Feb 18 2014	0.36%	24.7	68.9	4.7	Additional file 4: Figure S3
		May 29 2014	0.32%	30.6	58.6	4.3	
		Aug 27 2014	0.03%	25.6	39.4	3.3	
2	H1047R	Dec 03 2013	2.39%	149.6	96.8	5.7	Fig. 1a
		Jan 02 2014	3.93%	77.3	91.6	12.7	
		Jan 30 2014	4.39%	3082.1	100.6	13.9	
		March 03 2014	9.61%	3629.3	138.1	19.8	
31	H1047R	March 27 2014	0.19%	13.5	89.3	22.1	Fig. 1c
		Aug 29 2014	0%	0	30.7	6.2	
		Oct 02 2014	0%	0	28.8	5.1	
		Nov 03 2014	0%	0	17.5	4.4	
11	E545K	March 06 2014	27.19%	9274.2	86	108	Additional file 4: Figure S2
		April 09 2014	6.61%	235.9	66	86	
3	E545K	Dec 02 2013	4.23%	412.5	57.6	2.8	Fig. 2
		March 12 2014	3.07%	1839.5	60.8	3	
		Sep 10 2014	1.97%	165.1	114.6	15.1	
		Dec 15 2014	0.76%	143.5	179.5	9	
6	E542K	Feb 10 2014	0.24%	63.30	115.6	3.9	Additional file 4: Figure S4
		Apr 04 2014	0%	0	88.5	2.9	
		Sep 05 2014	0%	0	45	1.7	

### Concordance between imaging assessments and PIK3CA mutation quantification in plasma

**Fig. 1:**
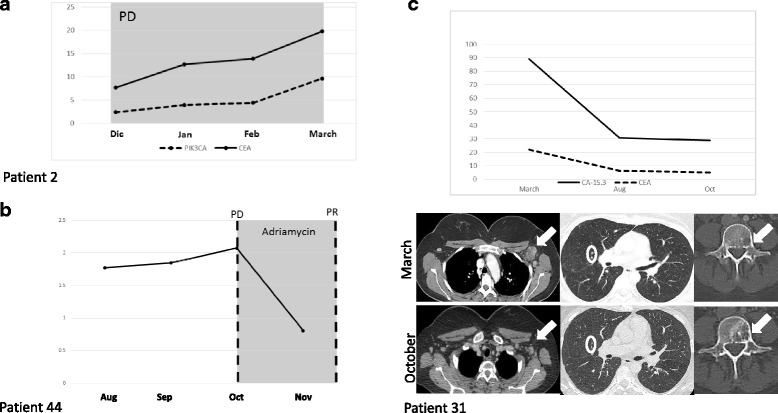
Panel (**A**) shows plasma levels of H1047R PIK3CA mutation (%) on cfDNA quantified by dPCR and CEA (ng/ml) tumor marker across four time points (months) (Pearson correlation coefficient =0.92) from patient 2, PD = progressive disease. Panel (**B**) shows plasma levels of H1047R PIK3CA mutation (%) on cfDNA across four time points (months). Disease status as ascertained on a CT-scan at two time points is marked with a discontinuous line. The use of adryamicine based chemotherapy is indicated by *gray shading*. As shown, PIK3CA mutation quantification increased until October when the patient 44 was diagnosed as having a progressive disease (PD). Subsequently, the patient received a different line of palliative treatment. PIK3CA mutation significantly decreased from 2.08% (CI 1.768% – 2.429%) to 0.81% (0.487% – 1.342%) correlating with the partial response (PR) assessed by CT-scan. Panel (**C**) shows the plasma levels of tumor markers 15–3 (U/ml) and CEA (ng/ml) and CT-scans showing the bone metastasis, lung metastases and the axillary adenopathy from patient 31. In this case the H1047R mutation quantification went down to 0% over the course of treatment (from 0.19 to 0%) correlating with tumor markers, the visceral metastases (*white arrow*) and the adenopathies (*circle*) although the bone metastasis increased significantly in size (*white arrow*)

**Fig. 2:**
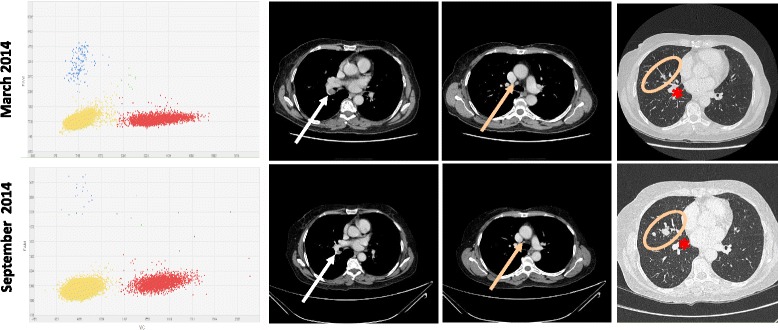
E545K assay scatter plots from patient of Patient 3 plasma samples and the corresponding CT scan images showing the hilar and mediastinal adenophaties and the lung metastases (*arrows* and *circles*). The asterisk indicates the lung hilum vessels

As an exploratory analysis, in the aforementioned 8 patients we compared the performance of PIK3CA assays with measurable disease (as defined by RECIST criteria). The median follow-up was 7.5 months. Overall, changes in ctDNA were in concordance with treatment responses observed in imaging. Partial responses were recognized in two patients (patient 25 and patient 44) during the follow-up period. In both cases, a reduction of mutant allele fraction in plasma samples was evidenced (Additional file 4: Figure S1 and Fig. 1b respectively). Similarly, progressive disease (PD) was documented in 4 women (patient 44, patient 2, patient 31, patient 3) during the study. An Increase in PIK3CA mutant allele fraction reflected progressive disease in 2 patients (patient 2 represented in Fig. 1a and patient 44 represented in Fig. 1b). Noteworthy, in one case (patient 31), although H1047R mutation quantification went down to 0% over the course of treatment (from 0.19 to 0%; Table 2) correlating with tumor markers CA15.3 and CEA variations and with the decrease in size of the pulmonary metastases and the axillary lymphadenopathy, the contrast-enhanced CT scan from the same period of time demonstrated that the lytic bone metastases increased in size (Fig. 1c). Similarly, in case of Patient 3, the hiliar and mediastinal adenopathies decreased in size from 13 to 8 mm to 7 and 4 mm respectively, correlating with E545K mutation quantification that significantly decreased from 3.07% (CI: 2.71% – 3.465%) to 1.97% (CI: 1.343% – 2.88%) (Table 2) in the corresponding plasma samples. However, the pulmonary metastases increased in number and size during the same period of time correlating with tumor markers quantification (Fig. 2). Finally, stable disease was documented in two patients (patient 15 and patient 6) in which tumor circulating free DNA fluctuated in the same way that tumor markers fluctuated (Additional file 4: Figure S3 and Figure S4).

## Discusion

In this study, we have tested the feasibility of array-based digital PCR to detect and quantify tumor specific mutations in plasma samples and we have documented the correlation between PIK3CA mutation quantification and tumor responses assessed by RECIST criteria.

Several groups [6, 18–21] have reported the utility of cfDNA as a source for tumor mutation detection. As described by others, we found a good agreement (K = 0.798) between plasma and FFPE samples when assessing PIK3CA mutation status in advanced patients, using array-based dPCR. However, in our experience the agreement was lower when early stages patients were included. Consistent with this, Oshiro et al have recently reported that PIK3CA mutations were detected in only 22.7% of serum samples from early-stage breast cancer patients with tumors harboring a PIK3CA mutation using array-based dPCR. Of important note, in that study, the authors proved that the sensitivity of the assay was 0.01% [22]. In the same way, Bettegowda C et al have reported ctDNA was detectable in >75% patients with advanced breast cancer disease whereas the frequency of cases with detectable ctDNA was 50% in early stages [23]. On the contrary, other researchers have reported a high agreement between liquid and solid biopsy for PIK3CA mutation status assessment in early stages using droplet digital PCR [19]. Nevertheless, the sample size of our study was limited and larger sized cohorts are needed to clarify this issue. To our knowledge, there is no data comparing the aforementioned methodologies (droplet vs array). Such studies would be of particular interest in order to determine the best strategy for biomarker testing to guide targeted cancer therapies using liquid biopsy.

The evaluation of tumor response to treatment that identifies patients early on that do not benefit from therapies remains a public health challenge. The capacity of serial monitoring of ctDNA to track tumor burden has been previously addressed by several researchers [6, 13, 24]. In this way, Dawson SJ et al analyzed PIK3CA mutation quantification in 13 cases [6]. According to the authors, PIK3CA mutation quantification correlated with changes in tumor burden. Similarly, we found that in 6 out of 8 cases the level of PIK3CA mutations correlated with treatment responses according to RECIST criteria. In the two remaining cases, the discordance could be reflective of tumor evolution. Indeed, the radiology evaluation of the disease revealed a different sensitivity of the metastatic lesions to treatment, highlighting the issue of heterogeneity within advance disease. Similarly, Higgins [18] et al reported a 27.5% discordance among 51 patients with recurrent metastatic disease prospectively tested by BEAMing in blood compared with standard sequencing of archival tissue. Yet, our findings were consistent with responses seen on imaging as we could impute the lesions that most probably harbored the PIK3CA mutation. It is well known that breast cancer is a heterogeneous disease and that molecular profile of cancer can change over time [25, 26]. This fact might limit usefulness of ctDNA to monitor response to treatment, especially when the mutation tracked is not targeted by therapy.

In any case, our results suggest that changes in mutant allele fraction in liquid biopsy should be analyzed together with imaging data in order to make mutation analysis informative.

## Conclusions

In this study, we have shown that fluctuations in the fraction of PIK3CA mutant allele always correlated with changes in lesion size seen on images, although not always with treatment responses, as defined by RECIST criteria. Our findings suggest that information provided by oncogenic mutation quantification in plasma samples could be limited by tumor heterogeneity in advanced disease and should be evaluated together with radiographic imaging.

## Additional files

Additional file 1: Clinical and pathological variables. (XLSX 16 kb)

Additional file 2: Limit of detection and limit of quantification estimation according to ICH guidelines. (XLSX 30 kb)

Additional file 3: Performance of PIK3CA assays on cfDNA. Data from sample chips are displayed in a scatter plot based on color of FAM and VIC events. Plots A, C and E correspond to a negative samples. Plots B, D and F correspond to positive samples for the H1047R, E545K and E542K mutation respectively. The mutation is labeled with FAM (blue data points) whereas wild-type is labeled with VIC (red data points). Yellow cluster represent the no amplification cluster. (PPTX 153 kb)

Additional file 4: Serial plasma PIK3CA mutation levels and treatment outcome, assessed according to RECIST criteria v.1.1. SD = stable disease, PR = partial response, PD = progressive disease of individual cases. (PPTX 78 kb)

## Abbreviations

cfDNA: Circulating free DNA
dPCR: Digital polymerase chain reaction
MAF: Mutant allele fraction
FFPE Formalin-Fixed: Paraffin-embedded
ER: estrogen receptor

## Footnotes

1 For Research Use Only. Not for use in diagnostic procedures.
