# Supplementary figures and images for: Tumor burden monitoring using cell-free tumor DNA could be limited by tumor heterogeneity in advanced breast cancer and should be evaluated together with radiographic imaging
Source: BMC Cancer. 2017 Mar 22;17:210. doi: 10.1186/s12885-017-3185-9 (PMC5362993; doi:10.1186/s12885-017-3185-9)

## Slide 1
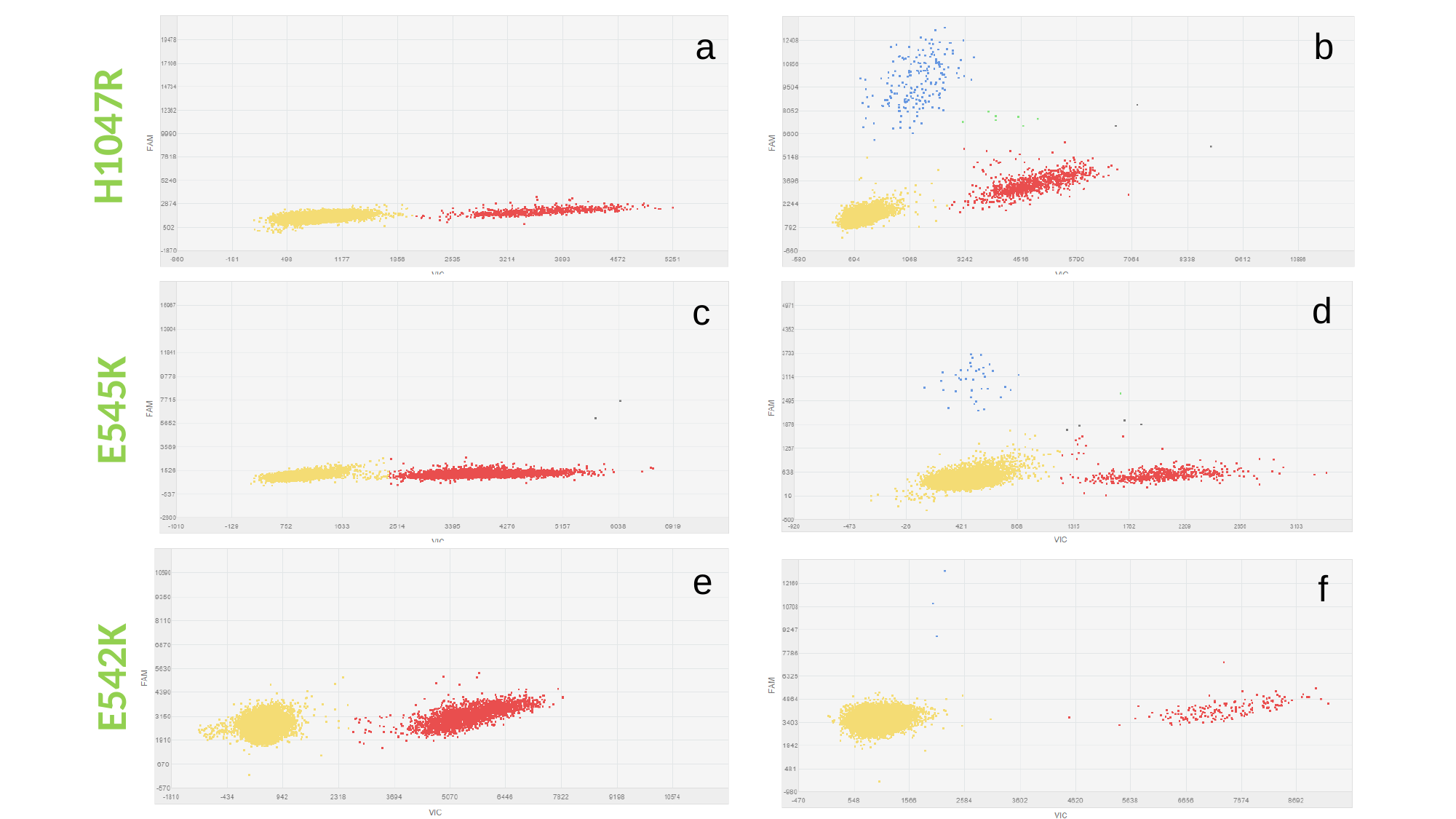

b
a
H1047R
A
B
d
c
E545K
e
f
E542K

Supplement: Supplementary file 3 — Performance of PIK3CA assays on cfDNA. Data from sample chips are displayed in a scatter plot based on color of FAM and VIC events. Plots A, C and E correspond to a negative samples. Plots B, D and F correspond to positive samples for the H1047R, E545K and E542K mutation respectively. The mutation is labeled with FAM (blue data points) whereas wild-type is labeled with VIC (red data points). Yellow cluster represent the no amplification cluster. (PPTX 153 kb) [file 12885_2017_3185_MOESM3_ESM.pptx]
