# Supplementary material for: Tumor burden monitoring using cell-free tumor DNA could be limited by tumor heterogeneity in advanced breast cancer and should be evaluated together with radiographic imaging
Source: BMC Cancer. 2017 Mar 22;17:210. doi: 10.1186/s12885-017-3185-9 (PMC5362993; doi:10.1186/s12885-017-3185-9)
Supplement: Supplementary file 4 — Serial plasma PIK3CA mutation levels and treatment outcome, assessed according to RECIST criteria v.1.1. SD = stable disease, PR = partial response, PD = progressive disease of individual cases. (PPTX 78 kb) [file 12885_2017_3185_MOESM4_ESM.pptx]

## Slide 1
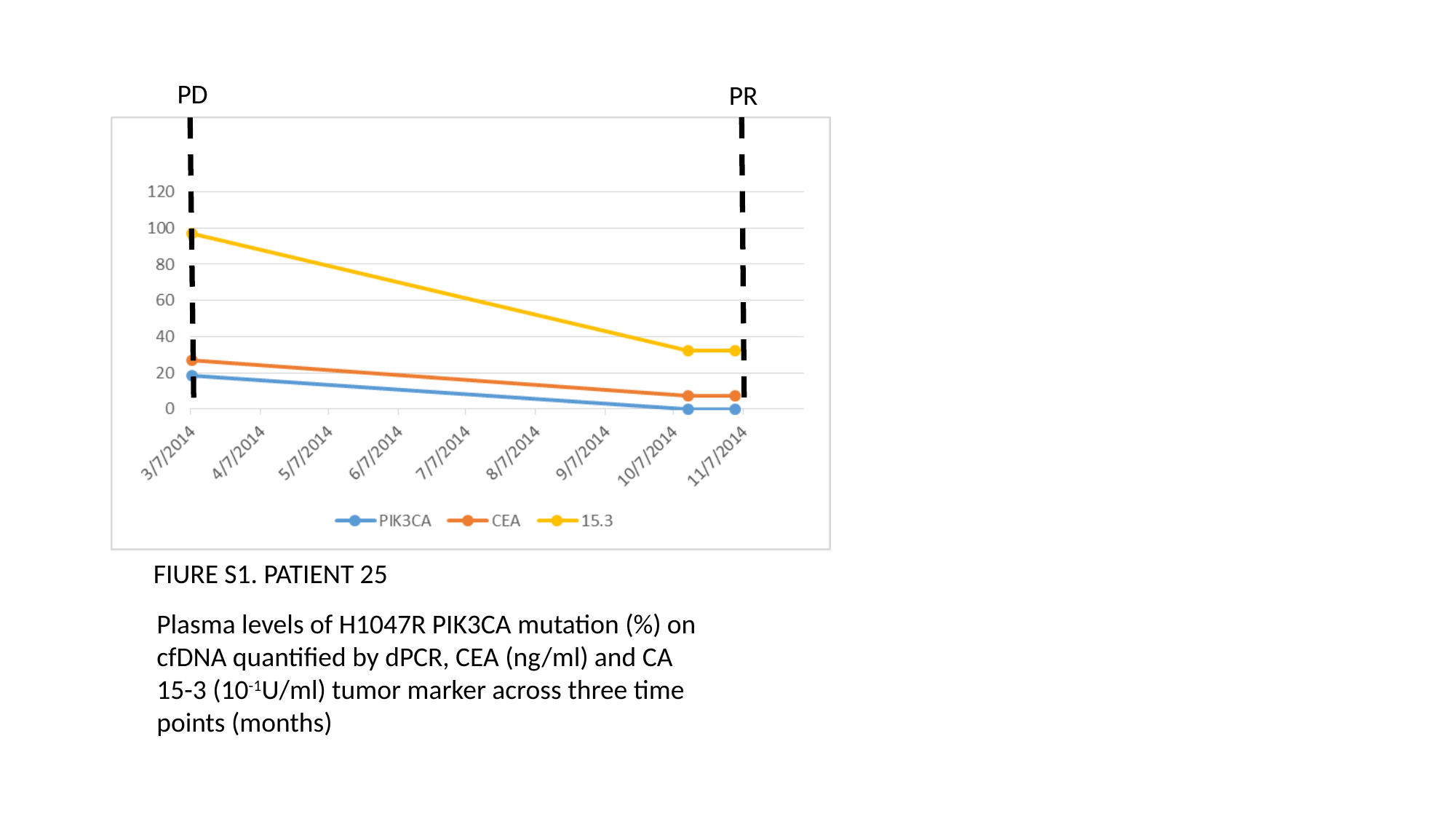

PD
PR
FIURE S1. PATIENT 25
Plasma levels of H1047R PIK3CA mutation (%) on cfDNA quantified by dPCR, CEA (ng/ml) and CA 15-3 (10-1U/ml) tumor marker across three time points (months)

## Slide 2
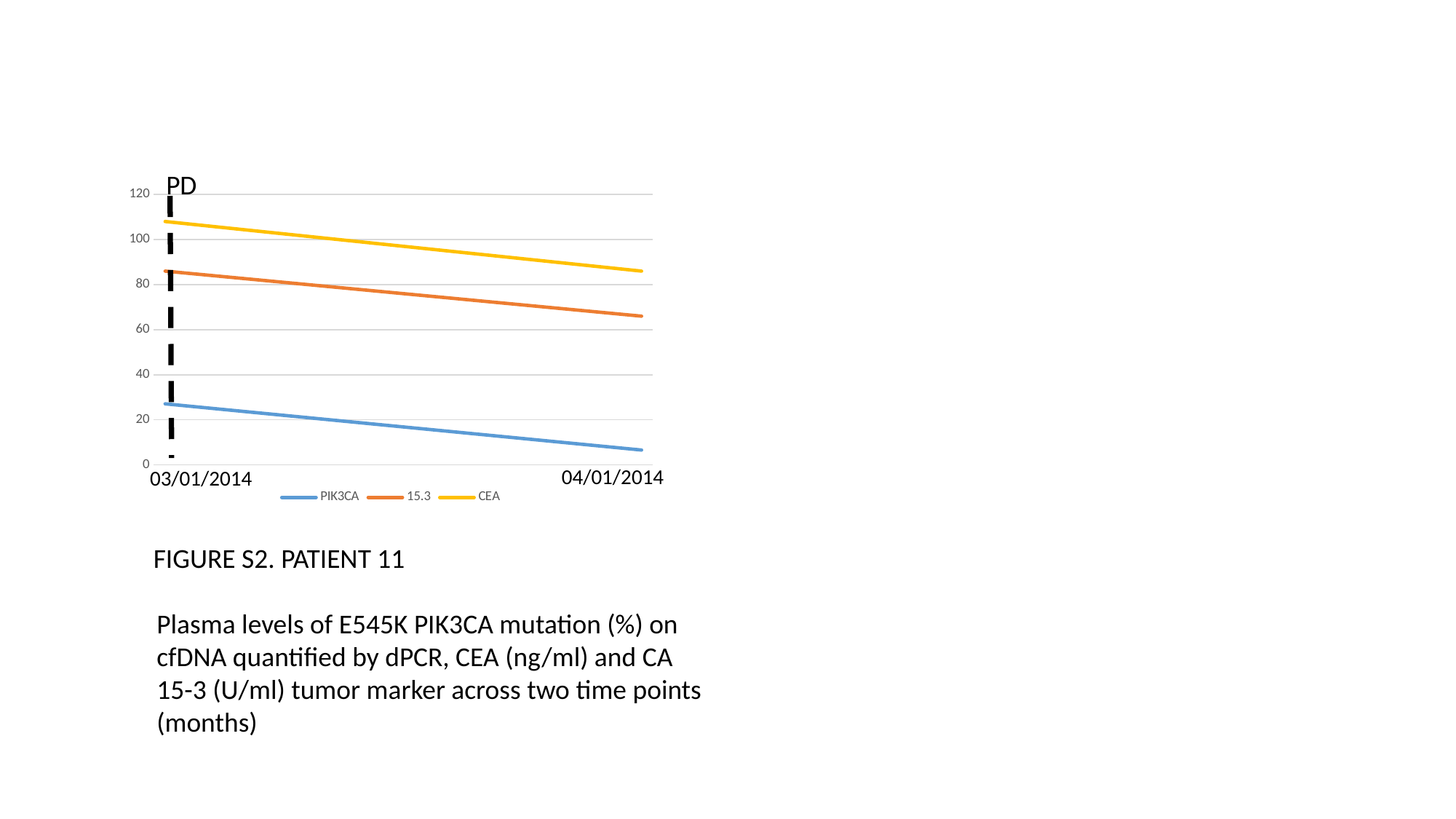

PD
### Chart
| Category | PIK3CA | 15.3 | CEA |
|---|---|---|---|
| 41704 | 27.1 | 86.0 | 108.0 |
| 41725 | 6.6 | 66.0 | 86.0 | 03/01/2014
 04/01/2014
FIGURE S2. PATIENT 11
Plasma levels of E545K PIK3CA mutation (%) on cfDNA quantified by dPCR, CEA (ng/ml) and CA 15-3 (U/ml) tumor marker across two time points (months)

## Slide 3
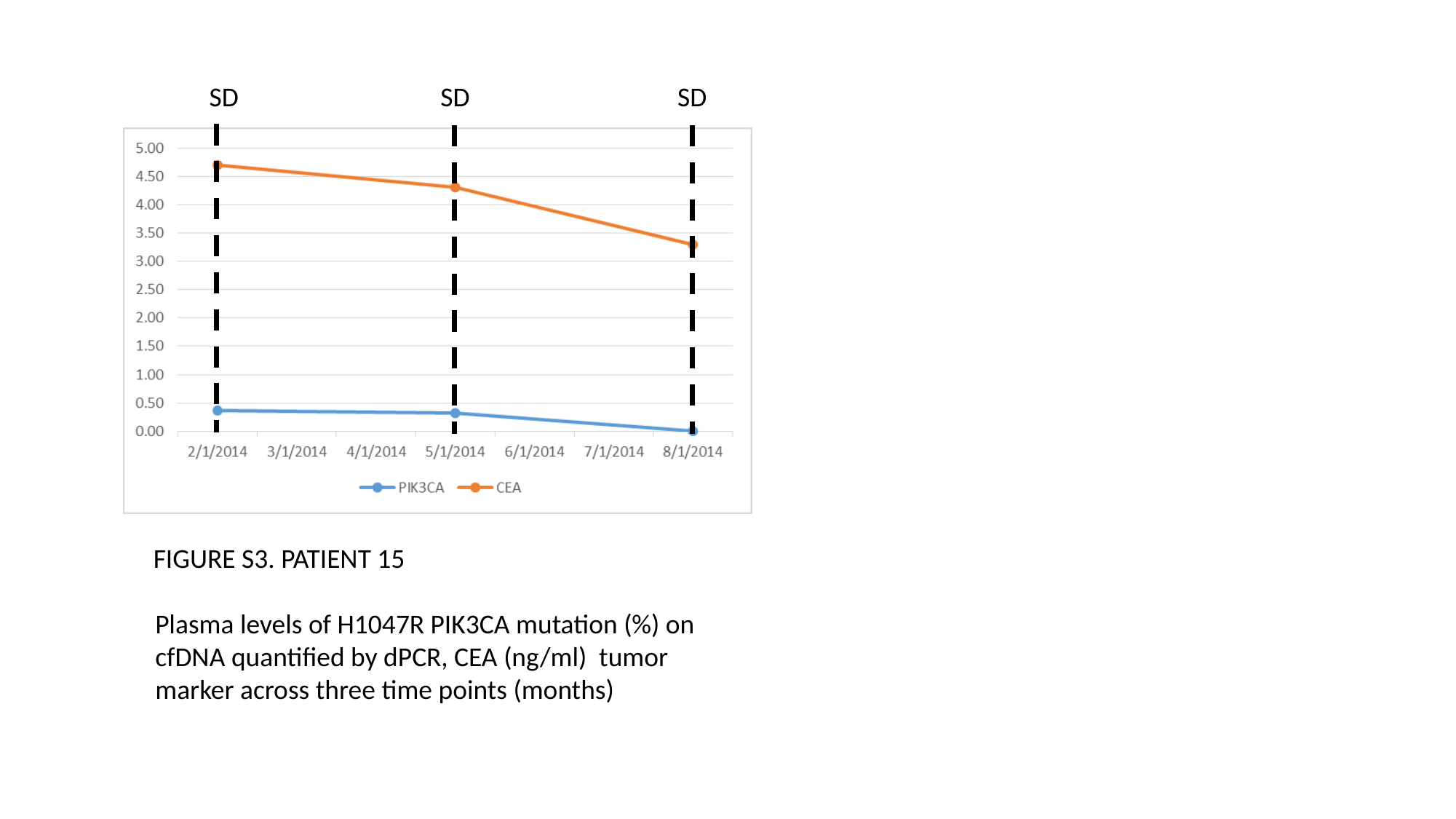

SD
SD
SD
FIGURE S3. PATIENT 15
Plasma levels of H1047R PIK3CA mutation (%) on cfDNA quantified by dPCR, CEA (ng/ml) tumor marker across three time points (months)

## Slide 4
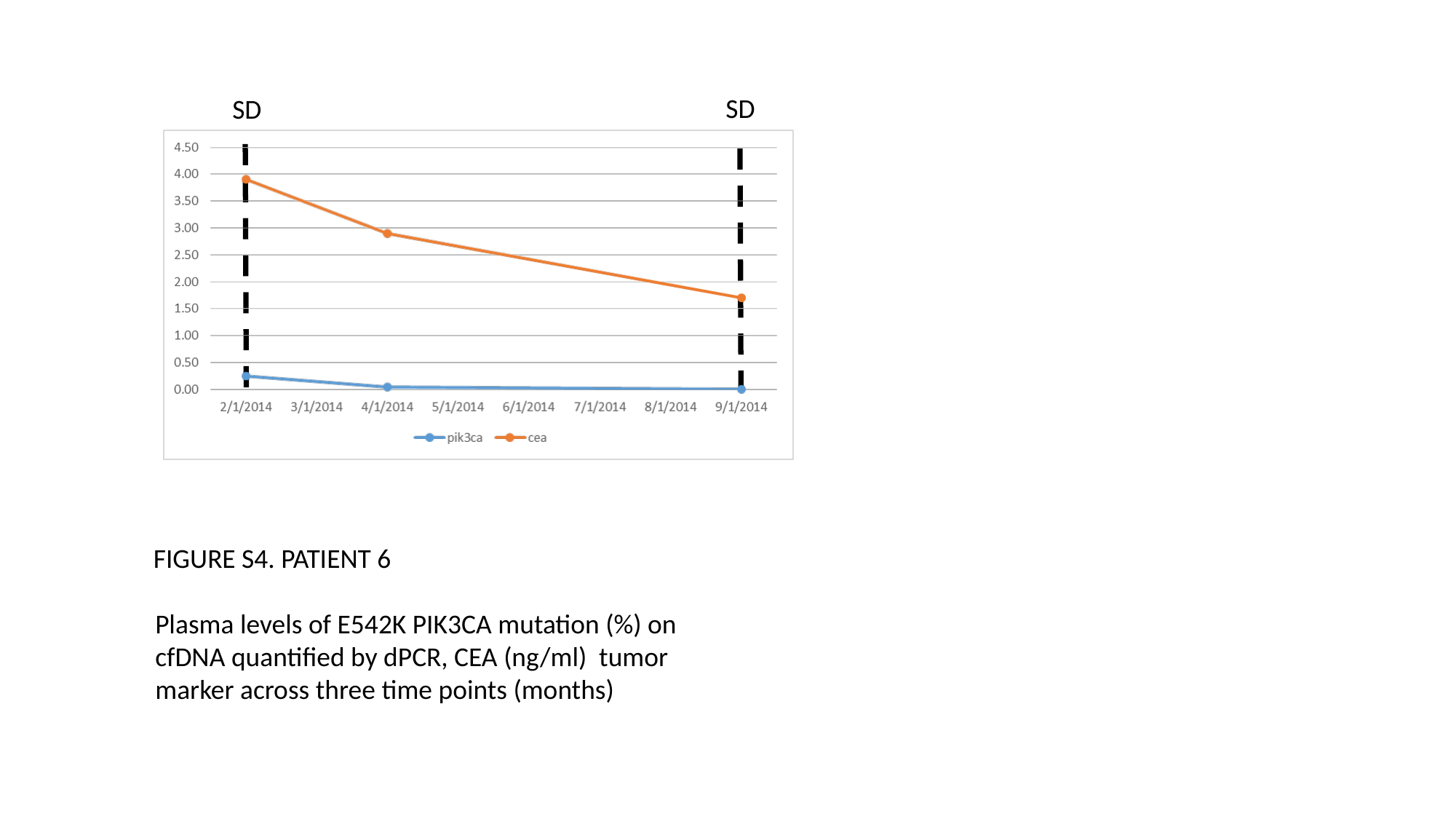

SD
SD
FIGURE S4. PATIENT 6
Plasma levels of E542K PIK3CA mutation (%) on cfDNA quantified by dPCR, CEA (ng/ml) tumor marker across three time points (months)
